# Brd4 Is Essential for IL-1β-Induced Inflammation in Human Airway Epithelial Cells

**DOI:** 10.1371/journal.pone.0095051

**Published:** 2014-04-23

**Authors:** Younis M. Khan, Paul Kirkham, Peter J. Barnes, Ian M. Adcock

**Affiliations:** 1 Airways Disease Section, National Heart & Lung Institute, Imperial College London, London, United Kingdom; 2 School of Applied Sciences, University of Wolverhampton, Wolverhampton, United Kingdom; Leiden University Medical Center, Netherlands

## Abstract

**Background:**

Chronic inflammation and oxidative stress are key features of chronic obstructive pulmonary disease (COPD). Oxidative stress enhances COPD inflammation under the control of the pro-inflammatory redox-sensitive transcription factor nuclear factor-kappaB (NF-κB). Histone acetylation plays a critical role in chronic inflammation and bromodomain and extra terminal (BET) proteins act as “readers” of acetylated histones. Therefore, we examined the role of BET proteins in particular Brd2 and Brd4 and their inhibitors (JQ1 and PFI-1) in oxidative stress- enhanced inflammation in human bronchial epithelial cells.

**Methods:**

Human primary epithelial (NHBE) cells and BEAS-2B cell lines were stimulated with IL-1β (inflammatory stimulus) in the presence or absence of H_2_O_2_ (oxidative stress) and the effect of pre-treatment with bromodomain inhibitors (JQ1 and PFI-1) was investigated. Pro-inflammatory mediators (CXCL8 and IL-6) were measured by ELISA and transcripts by RT-PCR. H3 and H4 acetylation and recruitment of p65 and Brd4 to the native *IL-8* and *IL-6* promoters was investigated using chromatin immunoprecipitation (ChIP). The impact of Brd2 and Brd4 siRNA knockdown on inflammatory mediators was also investigated.

**Result:**

H_2_O_2_ enhanced IL1β-induced IL-6 and CXCL8 expression in NHBE and BEAS-2B cells whereas H_2_O_2_ alone did not have any affect. H3 acetylation at the *IL-6* and *IL-8* promoters was associated with recruitment of p65 and Brd4 proteins. Although p65 acetylation was increased this was not directly targeted by Brd4. The BET inhibitors JQ1 and PFI-1 significantly reduced IL-6 and CXCL8 expression whereas no effect was seen with the inactive enantiomer JQ1(-). Brd4, but not Brd2, knockdown markedly reduced IL-6 and CXCL8 release. JQ1 also inhibited p65 and Brd4 recruitment to the *IL-6* and *IL-8* promoters.

**Conclusion:**

Oxidative stress enhanced IL1β-induced IL-6 and CXCL8 expression was significantly reduced by Brd4 inhibition. Brd4 plays an important role in the regulation of inflammatory genes and provides a potential novel anti-inflammatory target.

## Introduction

Chronic inflammation is a core component of COPD and is associated with activation of the NF-κB signalling pathway particularly in patients with GOLD stage I-III disease [1], [2]. Elevated expression of oxidants, either derived from activated immune and structural cells or from cigarette smoke, result in the high degree of oxidative stress which is found in the lungs of COPD patients [3]-[5]. Oxidative stress and inflammation are inseparably intertwined processes in these subjects. There is also a considerable evidence of oxidative stress entailed in the pathology of many other disorders, including aging, cancer, neurodegenerative and cardiovascular diseases [6], [7]. Corticosteroids are frequently used in the management of inflammation in COPD patients; however, they proved to be less effective in COPD patients [8], [9].

Abnormal histone acetylation (AcH) profiles have been linked to smoke exposure [10] and to relative corticosteroid unresponsiveness in COPD [11], [12]. DNA is tightly packed together with histones into structural units called nucleosomes. Each nucleosome is an octamer of four core histone proteins; H2A, H2B, H3 and H4 proteins with ∼146-base pair of DNA wrapped around and linked to H1 protein [13]. In transcriptionally active chromosomal regions, the chromatin unwinds allowing accessibility of transcription machinery. In contrast, the condensed heterochromatin is associated with gene suppression. This transition is achieved through reversible post-translational modifications (PMTs) such as acetylation, methylation and phosphorylation [14]. PTMs of histones play an important role in gene transcription and regulation and generally occur at histone tails [15]. Histone lysine (K) acetylation (AcK) signals the recruitment of basal transcriptional co-activators to the promoter regions of inflammatory and immunoregulatory genes [16], [17]. Histone acetyltransferases (HATs) acts as writers and catalyse the addition of acetyl group to lysine residue in histone tails whereas histone deacetylases (HDACs) serve as erasers [18], [19]. Acetylated histones are recognised by the bromodomain and extra-terminal (BET) proteins that are considered as readers of acetylated histones and associated with the regulation of several genes involved in cellular proliferation, cell cycle progression and apoptosis [20], [21]. The BET proteins consists of Brd2, Brd3, Brd4 and testis-specific Brtd protein which all contain dual bromodomains at N-terminal regions and recognise AcK and conserved extra-terminal (ET) at C-terminal site which interacts with chromatin modifying proteins [20], [22]. Brd4 forms a complex with positive transcription elongation factor b (p-TEFb) and RNA polymerase II (RNA pol II) at the transcription start site (TSS) to transduce the AcK signal to drive gene expression [23], [24].

Recent studies have implicated Brd2 and Brd4 in the regulation of inflammatory genes in murine bone marrow-derived macrophages (BMDMs) [25], . Zhang and colleagues have also shown that BET inhibition results in down-regulation of a subset of lineage-specific genes in human CD4+ T-cells [27]. In addition, BET inhibitors have been reported to affect NF-κB-mediated gene expression in renal tubular cells [28], HEK293 and HepG2 cells [29]. In some instances, this reflected targeting of the non-histone acetylated NF-κB p65 subunit by Brd2 rather than an effect of Brd2/4 on AcH [30]. JQ1, a small synthetic compound, has been shown to inhibit the binding of BET proteins to AcH, resulting in reduction of tumour in the mouse model of NUT midline carcinoma [31] and proliferation of c-Myc-dependent proliferation of cancer cells [32]–[34]. Similarly, PFI-1, another Brd4 inhibitor, has been shown to have anti-proliferative effects on leukemic cells lines and abrogates clonogenic growth [35]. However, the anti-inflammatory properties of these compounds yet to be demonstrated under conditions of acute oxidative stress-enhanced inflammation in human airway epithelial cells.

In this study we show that both JQ1 and PFI-1, but not the inactive enantiomer of JQ1 [JQ1(-)], can suppress the NF-κB-mediated induction of *IL-6* and *IL-8* in primary human airway epithelial (NHBE) cells and in BEAS-2B cells. This effect was mimicked by knockdown of Brd4 but not of Brd2 and associated with loss of AcH3 and p65 binding to the native gene promoters.

## Materials and Methods

### Cell culture and treatment

SV-40 transformed human bronchial epithelial cells (BEAS-2B) were obtained from the American Type Culture Collection (ATCC, VA, USA) and grown to 70% confluence in keratinocyte conditioned medium (Gibco, Paisley, and UK) at 37°C and 5% CO_2_. Prior to experimentation, cells were serum-starved overnight in medium without epidermal growth factor and bovine pituitary extracts. Cells were stimulated with IL-1β (1 ng/ml) in the presence or absence of H_2_O_2_ (100 µM). The expression of IL-6 and CXCL8 was also investigated in the presence or absence of the BET inhibitors (JQ1 and PFI-1) following stimulation with IL-1β alone or in combination with H_2_O_2_.

### Normal Human Bronchial Epithelial (NHBE) cells Tissue culture

Normal human bronchial epithelial cells (NHBE) of non-smoking subjects were obtained from Lonza (Lonza, Slough, UK) and grown in bronchial epithelial cells growth medium (BEGM) supplemented with growth supplements (SingleQouts kit, Lonza) as recommended by the manufacturer. Cells were passaged at passages 2–8 using the ReagentPack subculture kit (Lonza) following suppliers instructions. Cells were cultured until 80% confluent at 37°C and 5% CO_2._ Prior to experiments, monolayers of cells (70–80% confluence) were incubated in basal medium (supplement free) overnight. Cells were treated with BET inhibitors (JQ1 and PFI-1) prior to stimulation with IL-1β (1 ng/ml) in the presence or absence of H_2_O_2_ (100 µM). All experiments were performed at least four times.

### Cell viability

Cell viability was assessed using the methylthiazolyldiphenyl-tetrazolium bromide (MMT) assay as described previously [36] and the Aqua LIVE/DEAD Fixable dead cell stain kit (Invitrogen, Paisley, UK) exactly according to the manufacturer's instructions. For the former assay, cells (4×10^4^/well) were plated in 200 µl serum-free media overnight into 96-well culture plate before incubation with 1 mg/ml MTT solution for 30 mins. MTT solution was then removed and dimethyl sulfoxide (DMSO) was added to dissolve the formazan product to produce a purple solution. The absorbance was measured at 550 nm. The colour intensity was correlated with cell viability. In the latter assay, cells were collected from the plate by trypsinisation and neutralized with serum-free medium, washed and re-suspended in 1 ml PBS. 1 µl of Aqua LIVE/DEAD Fixable dead cell stain was added per sample and left on ice for 30 mins in dark. Cells were washed twice with cold PBS and resuspended in 1% BSA and PBS followed by flow cytometric analysis to distinguish between positive cells (heat-treated dead cells) and negative cells (alive).

### Cytokine ELISA

IL-6 and CXCL8 expression were quantified by sandwich ELISA (R&D Systems Europe, Abingdon, UK) according to the manufacturer's instructions.

### DCF assay for intracellular oxidative stress

Intracellular oxidative stress was detected as previously described [37]. Briefly, cells (4×10^4^/well) were plated into 96-well culture plate in serum-free media overnight before experimentation. Cells were washed with Krebs-Ringer-Hepes-glucose-glutamine buffer (KRH) on the day of experiment followed by incubation with dichlorofluorescine diacetate (100 µM; DCFH-DA) for 30 mins in loading medium. Cells were washed and incubated with KRH buffer with H_2_O_2_ (100 µM) for 2 hrs. The fluorescence from each well was measured with excitation and emission filter set at 485 nm and 530 nm, respectively.

### Nuclear and cytoplasmic extractions

Following treatments, cells were collected and nuclear and cytoplasmic proteins were extracted with nuclear extract kit (Active Motif, Carlsbad, California, USA) according to the manufacturer's instructions. The quality and purity of the subcellular fractionation was determined by immunoblotting using antibodies against cytoplasmic (β-actin) and nuclear (TATA binding protein) proteins to demonstrate standardization of this method.

### NF-κB activation assay

NF-κB activation after 2 hrs was measured in nuclear extracts with TransAM NF-κB activation kit (Active Motif) according to the manufacturer's instructions.

### Co-Immunoprecipitation (Co-IP)

Whole cell lysates were prepared by incubating cells with IP buffer (150 mM NaCl, 50 mM Tris-HCl pH 8, 0.5% Sodium deoxycholate, 0.5% NP40, protease inhibitors cocktail [Roche Applied Science, Burgess Hill, UK) for 30 mins on ice and centrifuged at 14000 rpm for 10 mins. 500 µg of protein extract was incubated with 5 µg of antibody Brd4 (Santa Cruz Biotechnology, Santa Cruz, California USA). The immune complex was incubated overnight on rotator at 4°C. Protein A/G-plus agrose beads (20 µl 50% slurry; Santa Cruz Biotechnology) were added to the complex and left on the rotator at 4°C for 2 hours. The complex was then washed using IP wash buffer (50 mM Tris-HCl pH 7.4, 0.5% (v/v) NP40, 150 mM NaCl) three times. Proteins were eluted from complex using 35 µl of elution buffer (50 mM Glycine, HCl pH 2.4) followed by addition of 3.5 µl of neutralising solution (2 M Tris pH 8, 1.5 M NaCl, 1 mM EDTA). Samples were run on SDS-PAGE gel.

### Western blotting

Nuclear and cytoplasmic extracts were fractionated by SDS-PAGE gel as previously described [38]. Proteins were transferred and immobilised on nitrocellulose membranes. Membranes were incubated with specific antibodies directed against NF-κB p65, β-actin, TATA-binding protein (All from Santa Cruz Biotechnology), acetylated p65-lysine 310 (Abcam, Cambridge, UK), Brd2 and Brd4 (Bethyl Lab, Montgomery, TX, USA). After washing, membranes were incubated with horseradish peroxidase-linked anti-rabbit immunoglobulin (DAKO Ltd, Ely, UK) and detected by enhanced chemiluminescence (ECL) (GE healthcare, Amersham, UK). The membranes were exposed to X-ray and the bands density was quantified using the GelDoc Imaging System (UVP, Upland, CA). This was standardised against the loading controls and the data were represented in graphs.

### Measurement of mRNA transcripts

Cells were treated as above and total RNA was isolated using an RNeasy mini kit (Qiagen, Crawley, UK) and the concentration of RNA determined using the absorbance ratio of 260 nm/280 nm by NanoDrop 2000c spectrophotometer (Thermo Fisher Scientific, Waltham, MA, USA). Single stranded cDNA was synthesized using AMV-reverse transcriptase (Promega, Southampton, UK) as described previously [39]. QPCR was performed in a Rotor-Gene 3000TM PCR machine (Corbett, Research, Cambridge, UK) using a QuantiTect SYBR Green PCR Kit (Qiagen) according to the manufacturer's instructions. The RT-PCR primers used were as follows: *Brd2* (NCBI: NM_005104.3)-F, 5′-GGGGTGGCAGTGCTGCTTTA-3′; *Brd2*-R, 5′-GCTCAGCTGCCGCTTCTCAT-3′; *Brd4* (NCBI: NM_014299.2)-F, 5′-CCACACTGCGTGAGCTGGAG-3′; *Brd4*-R, 5′-ATCTTGGAGGAGCCGGCAAT-3′; *GNB2L1* (NCBI: NM_006098.4)-F, 5′-CTCCGCCTCTCGAGATAAGACC-3′; *GNB2L1*-R, 5′-GCAAACTGGCCATCTGAGGA-3′; *IL6* (NCBI: NM_000600.3)-F, 5′-AGGAGACTTGCCTGGTGAAA-3′; *IL6*-R, 5′-GCTCTGGCTTGTTCCTCACT-3′; *IL8* (NCBI: NM_000584.3)-F, 5′-AGACAGCAGAGCACACAAGC-3′; *IL8*-*R*, 5′-ATGGTTCCTTCCGGTGGT-3′. The PCR data for each gene of interest was normalized to housekeeping gene (*GNB2L1*) and represented as fold change using the delta-delta CT (2-^ΔΔ*CT*^) method.

### Chromatin Immunoprecipitation (ChIP) assay

ChIP assay was carried out as previously described [38]. Briefly, epithelial cells (BEAS-2B) were treated with H_2_O_2_ (100 µM) in the presence or absence of IL-1β (1 ng/ml) or left untreated for 2 hrs. In some experiments, cells were pre-treated with JQ1 (500 nM) for 4 hrs. After treatments, protein-DNA complex were fixed by formaldehyde (1% final concentration) and immunoprecipitated with either an anti-p65 (5 µg, Santa Cruz Biotechnology) or an anti-Brd4 (5 µg, Bethyl Lab) antibody overnight. NF-κB p65 and Brd4 binding to the *IL-8* and the *IL-6* promoter were quantified by real-time QPCR using a QantiTech SYBR green PCR kit (Qiagen) on Rotor gene 3000 (Corbett Research). Immunoprecipitated DNA was quantified by qPCR and compared to the level of promoter specific DNA obtained at t = 0 which was given an arbitrary value of 1. *IL-8* [40], [41] and *IL-6* [42]-[44] primers have been previously published. Primer pairs of *IL-6* and *IL-8* were as follows: *IL-6*, forward, 5′-AGCACTGGCAGCACAAGGCAAAC-3′ and *IL-6*, reverse 5′-CAAGCCTGGGATTATGAAGAAGG-3′; and IL-8, forward, 5′-GGGCCATCAGTTGCAAATC-3′ and *IL-8*, reverse, 5′-TTCCTTCCGGTGGTTTCTTC-3′.

### siRNA transfection

BEAS-2Bs cells were seeded in 6-well plates at 0.3×10^6^ cells/well and transfected with ON-Target plus SMART pool Brd2 (L-004935-00-0005) or Brd4 (L-004937-00-0010) or control (D-001810-10-05) small interfering RNAs (siRNA) at 20 nM concentration (all from Dharmacon Thermo Scientific, Chicago, IL, USA) using HiPerFect transfection reagent (Qiagen) as described by the manufacturer. After 72 hrs, nuclear extracts were examined for Brd2 and Brd4 protein expression using Western blotting. Total mRNA was extracted and converted to cDNA and Brd2 and Brd4 expression were assessed.

### Confocal Microscopy

Epithelial cells were seeded on to 2-well chambered coverslips (Sigma-Aldrich, Poole, UK) and allowed to attach overnight at 37°C and 5% CO_2_ in basal media. Cells were fixed and permeabilized using 3% paraformaldehyde (PFA) with 0.5% Triton-X-100 (Sigma-Aldrich) for 10 mins following stimulations. Slides were incubated with blocking buffer (50% fetal calf serum (FCS) 0.1%TX-100) for 30 min, followed by an hour incubation with primary anti-acetyl-Histone 3 antibody (Milipore, Watford UK) diluted to 1∶300, followed by an hour incubation with fluorescein isothiocyante-conjugated secondary antibody (Dako, Ely, UK). Cells were also stained with Alexa Fluor 488 phalloidin (Invitrogen) for cytoplasmic (Cytoskeleton actin) and DAPI (4',6-Diamidino-2-Phenylindole, Dihydrochloride; Invitrogen) for nuclear staining. Coverslips were mounted onto slides and allowed to dry before quantifying fluorescence intensities with imaging software (Leica Confocal Software Lite, Heidelberg, Germany).

### Statistical analysis

Data are represented as mean ± standard error mean (SEM). Data were analysed by Student's *t* test for two sets of data or by one-way ANOVA/Dunn's multiple comparison test for comparing more than two sets of data. GraphPad Prism (La Jolla, CA, US) was used to evaluate the data. Differences were considered significant for *P* values of ≤0.05.

## Results

### Hydrogen peroxide (H_2_O_2_) induces intracellular oxidative stress and enhances inflammatory mediator expression

**Figure 1:**
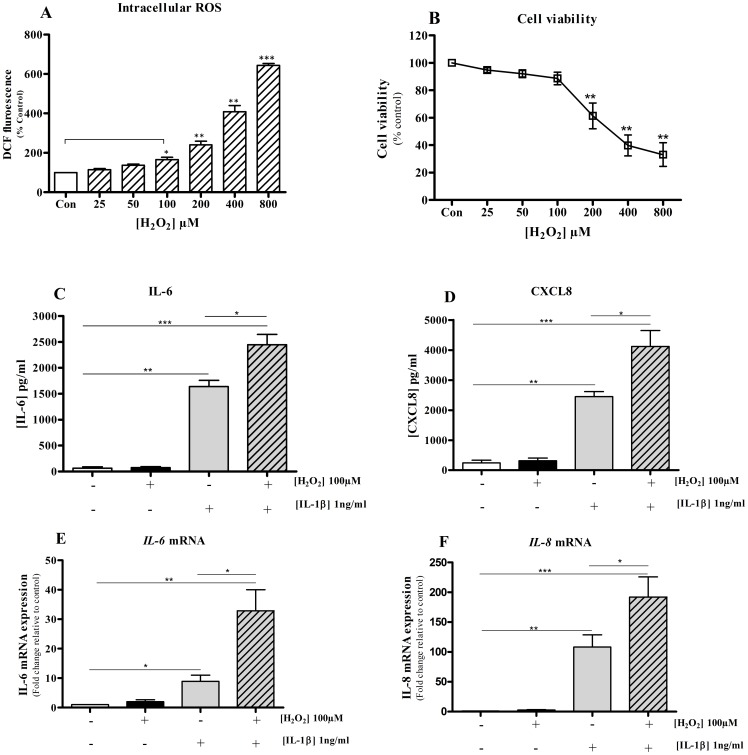
H_2_O_2_ induces intracellular ROS and enhances the inflammatory response. BEAS-2B cells were pre-incubated with DCFH-DA for 30 minutes in loading media followed by wash with KRH buffer. Cells were then treated with different concentrations of H_2_O_2_ in KRH buffer for 2 hours and intracellular ROS was measured (**A**). Cells were exposed to a range of concentrations of H_2_O_2_ for 2 hours and cell viability was assessed using MTT assay (**B**). Results are presented as mean ± SEM. N = 4. *p<0.05; **p<0.01; ***p<0.0001; when compared to basal level (control). BEAS-2B cells were treated with H_2_O_2_ for 2 hours in the absence or presence of IL-β stimulation (overnight) or left untreated as a control. IL-6 (**C**) and CXCL8 (**D**) protein levels in cell culture supernatants were quantified by ELISA. *IL-6* (**E**) and *IL-8* (**F**) transcript levels were quantified by comparative real-time PCR and were normalised by measuring *GNB2L1* transcript levels. Results are expressed as mean ± SEM of at least 4 independent experiments. * P<0.05; ** P<0.01; ***P<0.001 when compared to controls.

Exogenous H_2_O_2_ (0–800 µM) enhanced intracellular ROS in BEAS-2B cells a concentration-dependent manner after 2 hours which reached significance at concentrations of 100 µM or above (**Fig. 1A**). However, at concentrations >200 µM there was a significant loss in cell viability (**Fig. 1B**) and H_2_O_2_ (100 µM) was selected for all future experiments. H_2_O_2_ alone had no significant effect on either IL-6 or CXCL8 release at 16 hrs; however, H_2_O_2_ significantly enhanced the production of IL-6 (**Fig. 1C**) and CXCL8 (**Fig. 1D**) release from IL-1β (1 ng/ml) co-stimulated cells. This effect was mirrored by changes in *IL-6* and *IL-8* mRNA expression (**Fig. 1E–F**).

### The expression of *IL-6* and *IL-8* are regulated by the NF-κB signalling pathway

**Figure 2:**
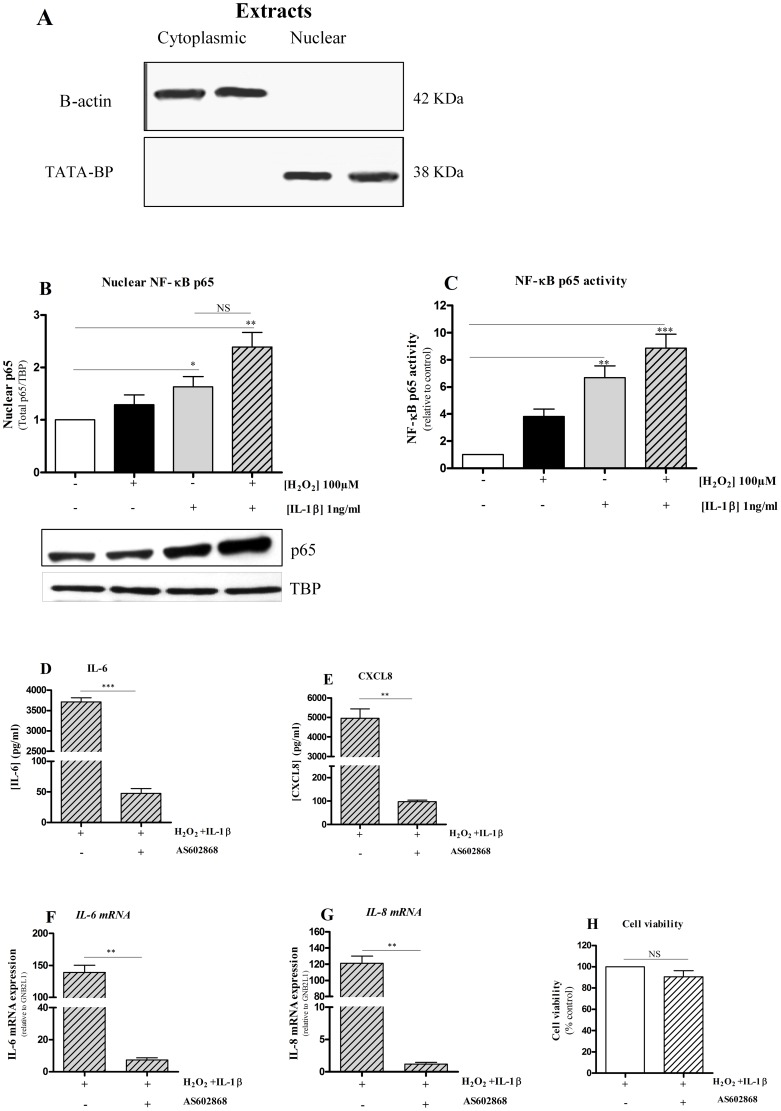
NF-κB p65 activates and translocates into the nucleus and IKK2 inhibitor AS602868 diminishes H_2_O_2_-enhanced IL-1β induction of cytokines. Cells were harvested and nuclear proteins were extracted from BEAS-2B cells after treatment with H_2_O_2_ (100 µM) in the presence (+) or absence (-) of IL-1β (1ng/ml) for 2 hours. The quality and purity of the subcellular fractionation was determined by immunoblotting using anti-β-actin and TBP (A). Using Western blot analysis, NF-κB p65 nuclear protein was quantified (B). Densitometric analysis of each band is plotted above. NF-κB p65 DNA binding activity was measured using TransAM kit (C). Results are expressed as means ± SEM as ratio of NF-κB p65/TATA-binding protein (TBP) or relative to untreated cells. n = 4 independent experiments *p<0.05; **p<0.01; ***P<0.001 compared with unstimulated cells. Cells were pre-treated with AS602868 (5 µM, IKK2 inhibitor) followed by the treatment with H_2_O_2_ with or without IL-1β (1 ng/ml) for 16 hours. IL-6 (D) and CXCL8 (E) proteins were assayed by ELISA. The release of IL-6 and CXCL8 was completely inhibited in cells pre-treated with AS602868. Levels of *IL-6* (F) and *IL-8* (G) mRNA were quantified by real-time RT-QPCR and were normalised with respective to *GNB2LI* mRNA levels. The RT-QPCR findings are consistent with the ELISA data (H). AS602868 (5 µM) did not affect cell viability using MTT assay. Results are expressed as mean ± SEM. n = 4. ** P<0.01; ***P<0.001 when compared to H_2_O_2_+IL-1β alone.

The subcellular extraction process was demonstrated to be highly reproducible with little or no cross-contamination of cytoplasmic and nuclear extracts using Western blotting (Fig. 2A). H_2_O_2_ alone had no significant effect on p65 nuclear translocation after 2 hrs whereas it enhanced IL-1β-induced p65 nuclear import (Fig. 2B). IL-1β significantly enhanced p65 DNA binding to a consensus κB-response sequence (5′-GGGACTTTCC-3′) 7-fold and this was further increased (up to 9-fold greater than baseline) by pre-treatment with H_2_O_2_ (Fig. 2C). In contrast, H_2_O_2_ alone had no significant effect on NF-κB p65 binding activity (Fig. 2C). Furthermore, IL-1β+H_2_O_2_-induced release of both IL-6 and CXCL8 proteins (Fig. 2D, E) and the expression of *IL-6* and *IL-8* mRNA (Fig. 2F, G) was completely suppressed by the selective IKK2 inhibitor AS602868 [45], [46]. Cell viability was not affected by AS602868 (Fig. 2H).

### Effect of IL-1β and H_2_O_2_ on p65 acetylation and association with Brd4

**Figure 3:**
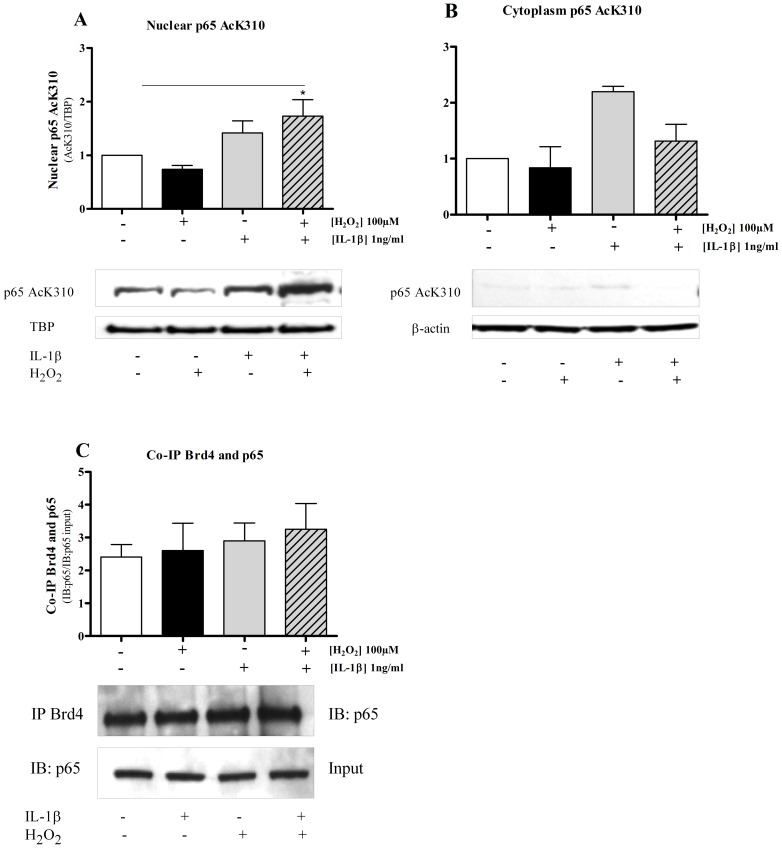
NF-κB p65 acetylation and association with Brd4 protein. (**A**) BEAS-2B cells were stimulated with H_2_O_2_ in the presence (+) or absence (-) of IL-1β (1 ng/ml) for 2 hours, nuclear (**A**) and cytoplasmic (**B**) extracts were fractioned by Western blot and membranes were probed with anti-acetylated NF-κB p65 antibody. The blots show that acetylated-310 (Ac310) NF-κB p65 is predominantly found in the nucleus when compared with the cytoplasm. (**C**) Brd4 protein was immunoprecipitated from whole cell extracts following treatments and separated by SDS-PAGE and subsequently analysed by Western blotting using an anti-NF-κB p65 antibody. Each blot is representative of 3 independent experiments and densitometric analysis of each band is plotted as bar graph above it. TBP: TATA-binding protein; *p<0.05; **p<0.01 compared with control (unstimulated).

NF-κB p65 is subjected to post-translational modifications such as acetylation that modulate its activity [47], [48]. Western blot analysis revealed that acetylated NF-κB p65 is found predominantly in the nuclear compartment in IL-1β stimulated cells after 2 hrs with little in the cytoplasmic compartment after any treatment (**Fig. 3A, B**). Although, H_2_O_2_ alone did not induce p65 K310 acetylation, IL-1β-induced p65 acetylation was enhanced after IL-1β+H_2_O_2_ co-stimulation (**Fig. 3A**). This suggests that acetylation of NF-κB at lysine-310 is associated with maximal NF-κB activation and/or translocation into the nucleus [49]. Brd4 can interact with acetylated p65 [24], [28] as part of a complex with p-TEFb and RNA polymerase II [28],[30]. Using co-immunoprecipitation experiments we found that there was an association between acetylated p65 and Brd4 (**Fig. 3C**), however, neither H_2_O_2_ nor IL-1β or in combination affected this association. This suggests that acetylated p65-Brd4 association is not directly linked to ROS-induced enhancement of NF-κB function.

### NF-κB p65 and Brd4 are recruited to the native *IL-6* and *IL-8* promoters

**Figure 4:**
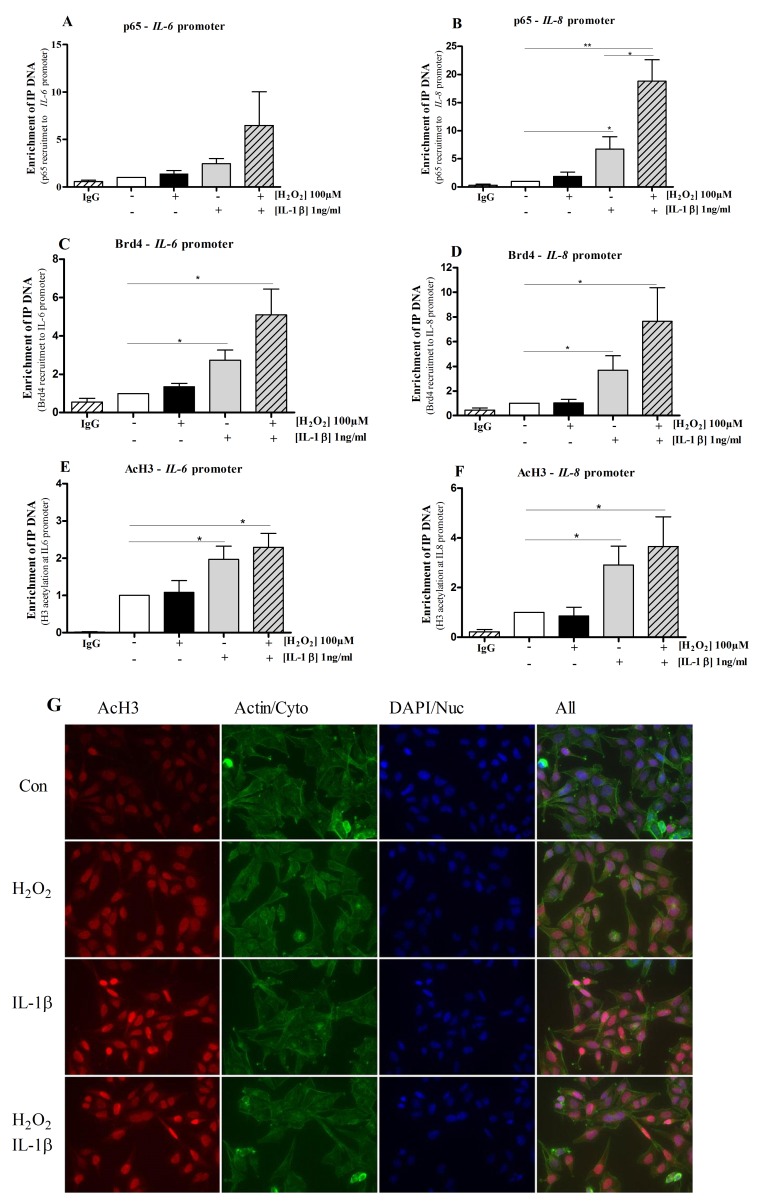
H3 acetylation, p65 and Brd4 binding to *IL-6* and *IL-8* κB promoter sites. Chromatin immunoprecipitation (ChIP) assays show that IL-1β induces p65 DNA binding to both *IL-6* (**A**) and *IL-8* (**B**) promoters. H_2_O_2_ by itself does not affect p65 DNA binding activity; however, when co-treated with IL-1β, the affinity is enhanced by 7-fold at *IL-6* promoter site and 20-fold at *IL-8* promoters. Brd4 is also recruited to the same κB promoter regions in the *IL-6* (**C**) and *IL-8* (**D**) promoters as p65. Histone 3 is acetylated at the *IL-6* (**E**) and *IL-8* (**F**) κB promoter sites following treatments. IgG is non-specific antibody used as a negative control. Furthermore, H3 acetylation was confirmed using confocal microscopy following IL-1β stimulation whereas H_2_O_2_ had no effect on AcH3 alone or in combination with IL-1β (**G**). Results are representative of at least 4 independent experiments.*p<0.05, **p<0.01 compared with control (unstimulated).

Following 2 hrs IL-1β treatment, p65 ChIP analysis (**Fig. 4A, B**) showed a marked enhancement in binding to κB elements within the native *IL-6* and *IL-8* promoters which was enhanced by co-stimulation with H_2_O_2_ (7- and 20-fold respectively). In contrast, H_2_O_2_ alone had no effect on p65 binding to either the *IL-6* or *IL-8* promoters. IL-1β also significantly increased binding of Brd4 to these κB sites in the *IL-6* and *IL-8* promoters (**Fig. 4C, D**). Again, this recruitment was augmented by co-stimulation with H_2_O_2_ and IL-1β at the *IL-6* (6-fold) and *IL-8* (8-fold) promoters compared to unstimulated cells (**Fig. 4C, D**). Pan-histone H3 acetylation at the *IL-6* and *IL-8* promoter κB sites was significantly elevated following IL-1β stimulation and slightly further increased with the addition of H_2_O_2_ although this did not reach significance (**Fig. 4E, F**). H_2_O_2_ alone did not affect Brd4 nor pan-histone H3 acetylation at these sites (**Fig. 4C-F**). Confocal analysis also confirmed an increased H3 acetylation in cells stimulated with IL-1β in comparison with unstimulated cells whereas H_2_O_2_ alone had little effect (**Fig. 4D**). We did not observe H4 acetylation at either of the promoters at the 2 hr time point studied, suggesting that H4 acetylation might be time- and gene-dependent.

### BET inhibitors reduce IL-1β induced inflammation

**Figure 5:**
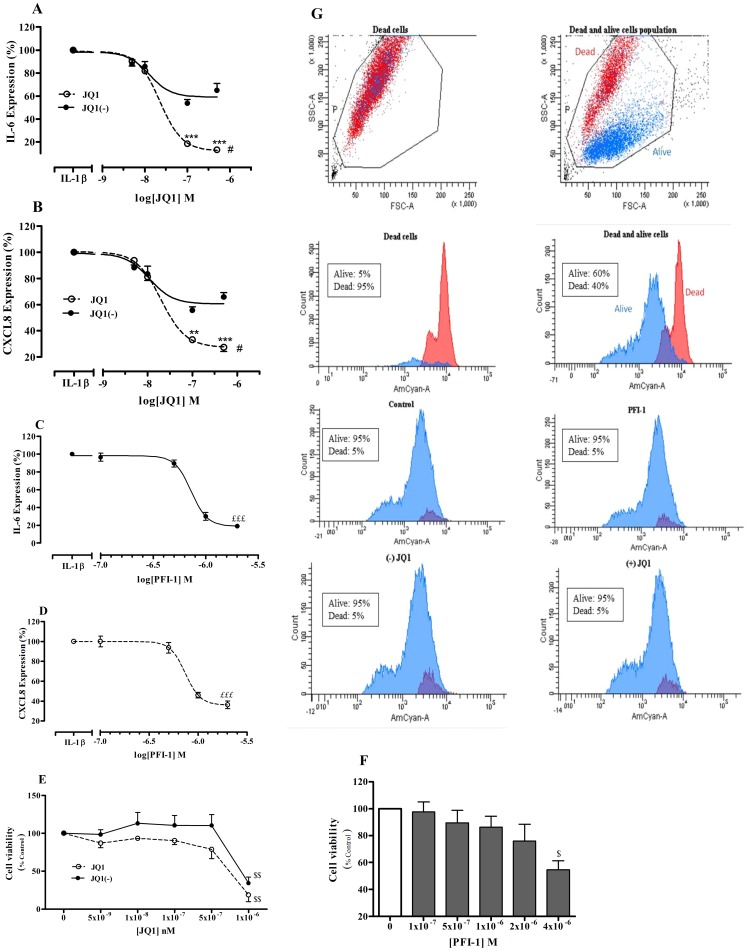
Concentration dependent reduction of IL-6 and CXCL8 by BET inhibitors. Cells were pre-treated with JQ1 and JQ1 (-) enantiomers (**A, B**) or PFI-1 (**C, D**) for 4 hours followed by IL-1β (1 ng/ml) for 16 hrs. IL-6 (**A, C**) and CXCL8 (**B, D**) proteins were assayed by ELISA. The effect of JQ1 and JQ1 (-) (**E**) and PFI-1 (**F**) on cell viability was assessed by MTT assay. n = 3 independent experiments. Points represent mean ± SEM **p<0.01;***P<0.001 compared with IL-1β stimulation. ^#^p<0.05 JQ1(-) versus JQ1. ^£££^p<0.001when PFI-1 compared with IL-1β stimulation. ^$^p<0.05; ^$$^p<0.01when compared to control. (**G**) Cells were heat treated at 90°C or left untreated, mixed together and stained with LIVE/DEAD Fixable Aqua stain then analysed by flow cytometry. Cells were checked with forward scatter detector (FSC) and side scatter detector (SSC) and analysed by density graph to check cell size and granularity. Fragmented cells were excluded from the study. Histogram shows separation of live cells (left) and dead cells (right). These parameters were used to assess cell viability following treatment with JQ1 (0.5 µM) and PFI-1 (1 µM) for 16 hours. DMSO/Control (<1%) alone, PFI-1, JQ1(-) or JQ1 resulted in only 5% decrease of overall cell viability. The data is representative of 3 independent experiments.

BEAS-2Bs cells were treated with JQ1 and its inactive enantiomer JQ1(-) at a range of concentrations (5×10^−9^−10^−6^ M) for 4 hours followed by IL-1β (1 ng/ml) stimulation for 16 hours. JQ1 inhibited IL-1β-induced IL-6 (**Fig. 5A**) and CXCL8 (**Fig. 5B**) release in a concentration-dependent manner with a maximal suppression of 86.8±2.0% (IL-6) and 72.5±3.1% (CXCL8) at 5×10^−7^ M. In contrast, JQ1(-) (5×10^−7^ M) had a non-significant suppressive effect on IL-1β-stimulated IL-6 (35.1±5.9%) and CXCL8 (34.1±3.3%) release (**Fig. 5A, B**). PFI-1, a structurally distinct BET inhibitor [50], also attenuated the release of both IL-1β-induced IL-6 (**Fig. 5C**) and CXCL8 (**Fig. 5D**) in concentration-dependent manner. The IC_50_ for PFI-1 inhibition of IL-1β-induced IL-6 protein levels was 7.3±4.2×10^-7^ M and a similar effect was seen for the inhibition of IL-1β-induced CXCL8 release (7.4±4.8×10^−7^ M). PFI-1 inhibited IL-1β-induced IL-6 and CXCL8 release with maximal suppression of (80.7±1.9%) and (63.6±3.8%), respectively.

Cell viability was significantly affected at the highest concentrations of JQ1 and JQ1(-) tested (10^−6^ M, **Fig. 5E**) and does not therefore account for the reduction in IL-6 and CXCL8 release seen at 5×10^−7^ M. High concentrations of PFI-1 (4×10^−6^ M) also significantly reduced cell viability and were excluded from subsequent experiments (**Fig. 5F**). These results indicated that PFI-1 has a similar efficacy as JQ1. We used PFI-1 at 1×10^−6^ M in subsequent experiments. The lack of effect of JQ1 and PFI-1 on cell viability as these concentrations was confirmed using FACS analysis and Live/Dead Aqua blue staining (**Fig. 5G**). Cells treated with DMSO (control), JQ1 (0.5 µM), JQ1(-) (0.5 µM) or with PFI-1 (1 µM) resulted in only 5% overall cell death with 95% cells being viable.

### BET inhibitors reduced oxidative stress-enhanced inflammation

**Figure 6:**
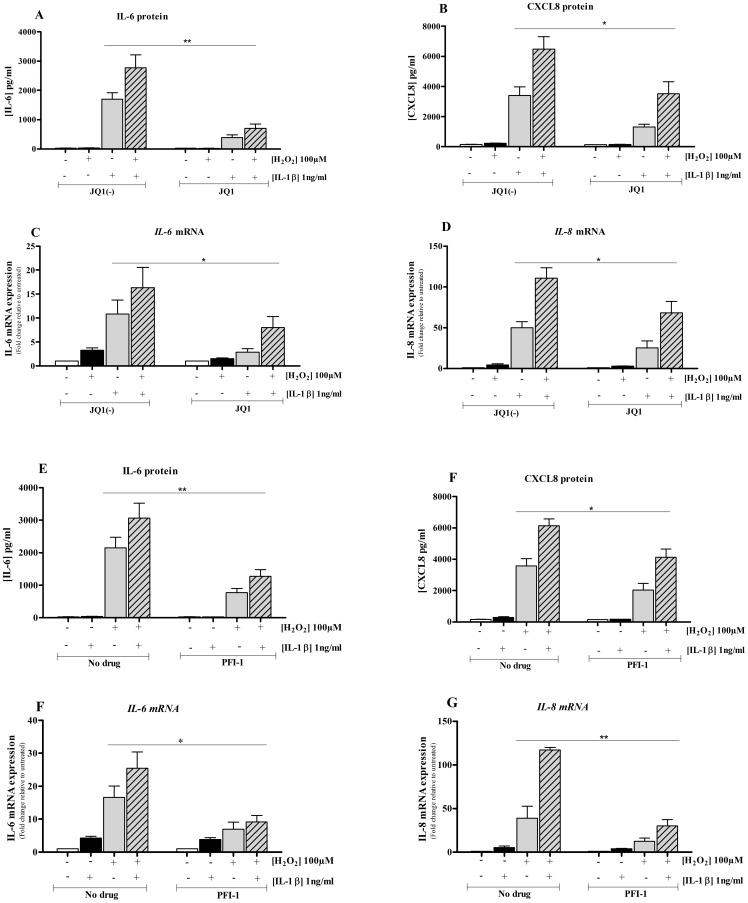
The BET inhibitors (JQ1 and PFI-1) reduce inflammatory mediator production. Cells were pre-treated with either JQ1 or JQ1(-) both at 500 nM for 4 hours followed by stimulation with H_2_O_2_ in the presence (+) or absence (-) of IL-1β (1 ng/ml) or both for 16 hours or left unstimulated. IL-6 (**A**) and CXCL8 (**B**) proteins were assayed by ELISA. *IL-6* (**C**) and *IL-8* (**D**) transcripts were quantified by RT-PCR. n = 4 independent experiments. Bar graph represents mean ± SEM *p<0.05, **p<0.01, when compared JQ1(-) with JQ1 treated cells. Under similar experimental conditions the effect of PFI-1 (1 µM) on IL-6 (**E**) and CXCL8 (**F**) proteins were assayed by ELISA. *IL-6* (**G**) and *IL-8* (**H**) mRNA levels were quantified by RT-QPCR. n = 4 independent experiments. Bar graph represent mean ± SEM *p<0.05, **p<0.01, when compared cells treated with or without PFI-1.

We demonstrated above that H_2_O_2_ (100 µM) alone has minimal effect on IL-6 and CXCL8 expression; however, it enhanced the expression of both cytokines (IL-6 and CXCL8) in IL-1β-induced cells. The release of IL-1β H_2_O_2_-induced IL-6 (**Fig. 6A**) and CXCL8 (**Fig. 6B**) proteins and *IL-6* (**Fig. 6C**) and *IL-8* (**Fig. 6D**) mRNA was markedly suppressed in JQ1 but not JQ1(-) treated cells. However, the levels of IL-6 and CXCL8 did not return to baseline. A similar effect was seen with PFI-1 which attenuated the release of IL-6 (**Fig. 6E**) and CXCL8 (**Fig. 6F**) proteins and *IL-6* (**Fig. 6G**) and *IL-8* (**Fig. 6H**) mRNA when compared with untreated cells. As with JQ1, mediator levels did not return to baseline in PFI-1 treated cells.

### The effects of JQ1 and PFI-1 are mimicked by Brd4, but not Brd2, knockdown

**Figure 7:**
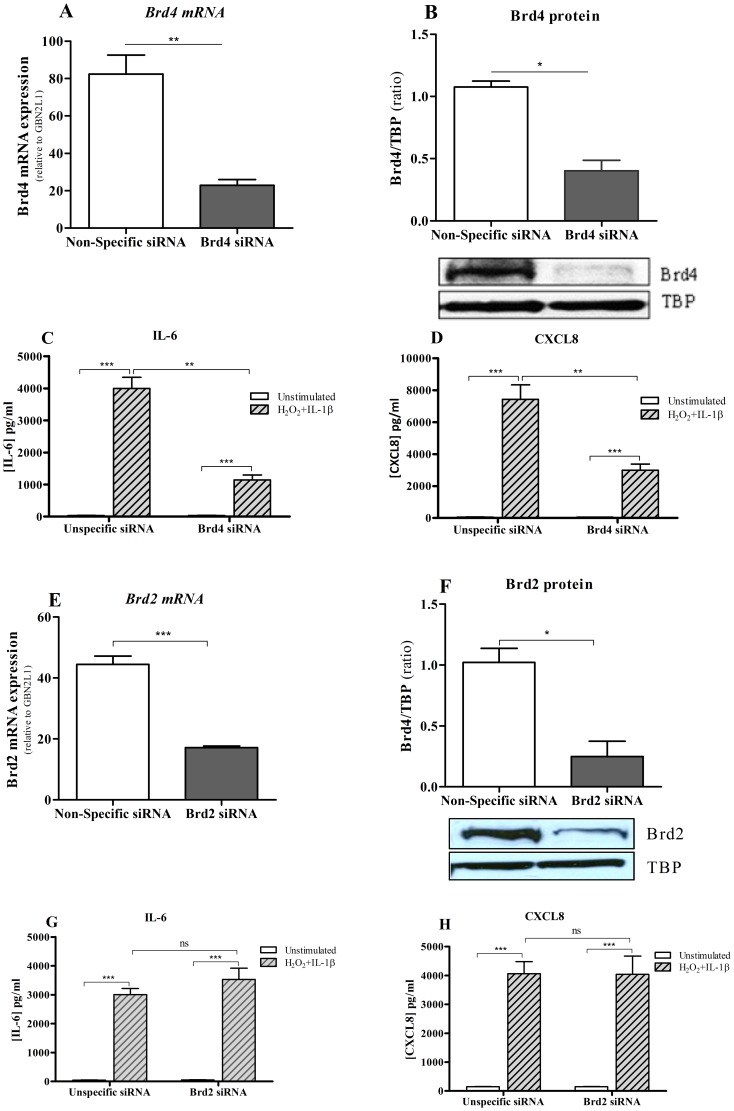
Knockdown of Brd4 reduces inflammation but not Brd2. Cells were transfected with either pooled Brd4 or Brd2 siRNAs (20 nM) or non-specific siRNAs (20 nM). 72 hours post-transfection Brd4 mRNA (**A**) was quantified by RT-QPCR and nuclear extracts were analysed (**B**) by immunoblotting using anti-Brd4 and -TATA-binding protein (TBP). IL-6 (**C**) and CXCL8 (**D**) proteins were measured by ELISA in post-transfected cells following stimulation with H_2_O_2_ and IL-1β together. n = 4 independent experiments. 72 hours post-transfection, Brd2 mRNA (**E**) was quantified by RT-QPCR and nuclear extracts were analysed (**F**) by immunoblotting using anti-Brd2 and TBP. Brd2 siRNA knockdown had no effect on either IL-6 (**G**) or CXCL8 (**H**) expression. n = 4 independent experiments. Results represent mean ± SEM *p<0.05, **p<0.01, ***p<0.001, non-specific siRNA compared to Brd2 or Brd4 siRNA.

To determine the specificity of the JQ1 and PF-1 effects, cells were transfected with Brd4 or non-specific small interfering RNAs for 72 hours resulting in significantly decreased expression of Brd4 mRNA (71.8±3.2%, p<0.05) (**Fig. 7A**) and protein 63.26±7.5%, p<0.05) expression (**Fig. 7B**). The release of IL-1β/H_2_O_2_-stimulated IL-6 (**Fig. 7C**) and CXCL8 (**Fig. 7D**) proteins was significantly suppressed in Brd4-knockdown cells in comparison with control non-specific siRNA transfected cells. The degree of suppression of IL-6 and CXCL8 was the similar to that seen in JQ1- and PFI-1-treated cells (**Fig. 6**). In contrast, although there is an 80% structural homology between Brd2 and Brd4 [51], [52] we were unable to show any effect of Brd2 knockdown on inflammatory gene expression. Following treatment of cells with Brd2 siRNA, there was a significant suppression of both Brd2 mRNA (**Fig. 7E**) and protein (**Fig. 7F**) expression, but no effect on IL-1β/H_2_O_2_-stimulated IL-6 (**Fig. 7G**) or CXCL8 (**Fig. 7H**) release. These data indicate that Brd4 is central to NF-κB-mediated induction of *IL-6* and *IL-8* expression and that JQ1 and PFI-1 preferentially target Brd4 and not Brd2 in airway epithelial cells.

### JQ1 inhibits p65 and Brd4 association at *IL-6* and *IL-8* promoters

**Figure 8:**
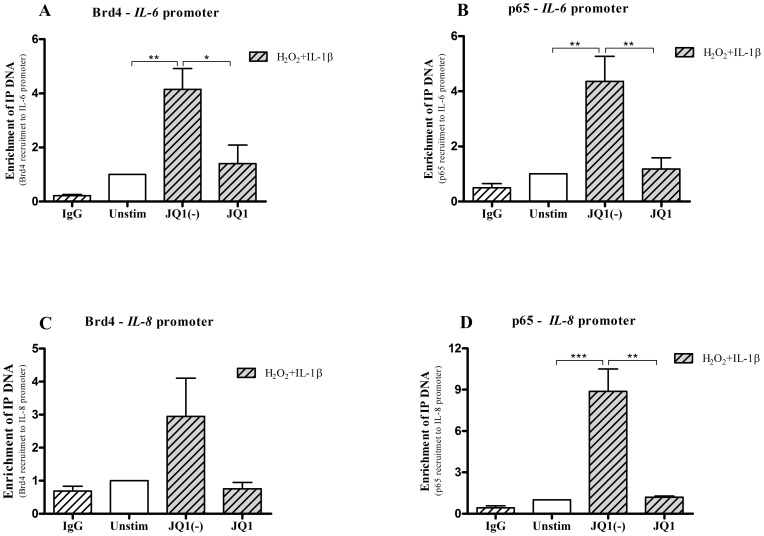
The effect of JQ1 on Brd4 and p65 binding to *IL-6* and *IL-8* promoters. Chromatin immunoprecipitation (ChIP) assay shows that IL-1β (1 ng/ml) and H_2_O_2_ (100 µM) induces Brd4 (**A**) and p65 (**B**) DNA binding to the *IL-6* promoter by 5-fold which is abolished in cells pre-treated with JQ1 (500 nM). Similarly, Brd4 (**C**) and p65 (**D**) DNA binding at the *IL-8* promoter is increased following H_2_O_2_ and IL-1β stimulation in JQ1(-) (500 nM) pre-treated cells by 10- and 4-fold. This binding is diminished in cells pre-treated with the active JQ1 (500 nM). Results are representative of at least 3 independent experiments.*p<0.05, **p<0.01, ***p<0.001 when compared with unstimulated cells.

ChIP analysis was used to investigate the mechanism of JQ1 at the p65 to the *IL-6* and *IL-8* promoters. Binding of Brd4 and p65 increased 5-fold at the *IL-6* promoter in H_2_O_2_+IL-1β stimulated cells when compared with unstimulated cells. This was not affected by the presence of the inactive enantiomer JQ1(-). However, JQ1 significantly reduced Brd4 (**Fig. 8A**) and p65 (**Fig. 8B**) binding at the *IL-6* promoters. Similarly, Brd4 and p65 association with the *IL-8* promoter increased 4-fold and 10-fold respectively in stimulated cells (H_2_O_2_+IL-1β). This association was abolished by JQ1 but was unaffected by JQ1(-) (**Fig. 8C, D**). The results also show that although the recruitment of p65 and Brd4 is completely abrogated at both the *IL-6* and *IL-8* promoters the expression of both *IL-6* and *IL-8* did not returned to baseline levels in JQ1 pre-treated cells. This suggests that post-transcriptional factors may also play a role in driving the expression of IL-6 and CXCL8 expression in these cells.

### JQ1 reduced oxidative stressed enhanced inflammation in human primary epithelial cells

**Figure 9:**
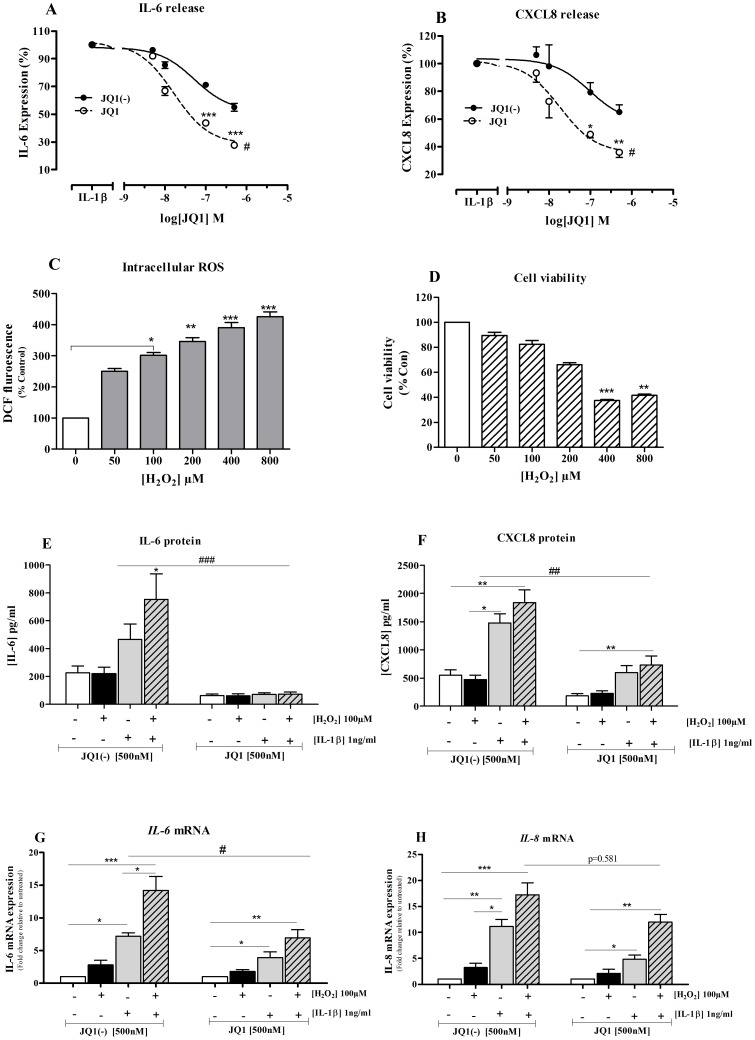
JQ1 reduces oxidative stress-enhanced IL-6 and CXCL8 expression in NHBE cells. NHBE cells pre-treated with JQ1 but not JQ1(-) (both at 5×10^−9^−10^−6^ M) reduced IL-1β induced release of IL-6 (A) and CXCL8 (B) in a concentration-dependent manner. Points represent mean ± SEM *p<0.05; **p<0.01;***P<0.001 when compared with IL-1β stimulation. ^#^p<0.05 JQ1(-) versus JQ1. NHBE cells were treated with a range of concentrations of H_2_O_2_ and intracellular ROS (**C**) and cell viability (**D**) were measured using DCFH-DA and MTT assay, respectively. Results are presented as mean ± SEM. N = 4. *p<0.05; **p<0.01; ***p<0.0001; when compared to untreated cells (control). Cells were pre-treated with either JQ1 or JQ1(-) both at 5×10^-7^ M for 4 hours followed by stimulation with IL-1β (1 ng/ml) in the presence (+) or absence (-) of H_2_O_2_ (100 µM) or both for 16 hours or left unstimulated. IL-6 (**E**) and CXCL8 (**F**) proteins were assayed by ELISA. *IL-6* (**G**) and *IL-8* (**H**) transcripts were quantified by RT-PCR. n = 5 independent experiments. Bar graph represents mean ± SEM *p<0.05; **p<0.01; ***p<0.001 when compared with controls. ^#^p<0.05; ^##^p<0.01; ^###^p<0.001 when comparing JQ1- with JQ1(-)-treated cells.

We repeated some key experiments in primary normal human bronchial epithelial (NHBE) cells from 4 different donors. We initially demonstrated that JQ1 had a significantly greater ability to suppress IL-1β-stimulated IL-6 (**Fig. 9A**) and CXCL8 (**Fig. 9B**) release than JQ1(-). JQ1 inhibited IL-1β-induced IL-6 and CXCL8 release in concentration-dependant manner with maximal suppression of 72.4±1.8% (IL-6) and 64.0±3.8% (CXCL8) at 5×10^−7^ M whereas the inactive enantiomer had much less effect on IL-6 (49.9±2.9) and CXCL8 (35.1±5.3) release.

We then examined the effect of H_2_O_2_ on NHBE cells function. H_2_O_2_ significantly induced intracellular ROS at concentrations >100 µM when compared to untreated cells (**Fig. 9C**) although this was associated with significantly reduced cell survival at concentrations >200 µM) (**Fig. 9D**). Therefore, NHBE cells were treated with 100 µM of H_2_O_2_ in the presence or absence of IL-1β in subsequent experiments. IL-1β-induced release of IL-6 (**Fig. 9E**) and CXCL8 (**Fig. 9F**) was further enhanced when co-treated with H_2_O_2_ which was mirrored by changes in *IL-6* (**Fig. 9G**) and *IL-8* (**Fig. 9H**) mRNA transcripts. There was no significant difference between IL-6 and CXCL8 expression between JQ1(-) and JQ1 treated cells under control conditions. JQ1 markedly attenuated the release of IL-1β/H_2_O_2_-induced IL-6 (**Fig. 9E**) and CXCL8 (**Fig. 9F**) proteins and *IL-6* (**Fig. 9G**) and *IL-8* (**Fig. 9H**) mRNA expression when compared with JQ1(-) treated cells.

## Discussion

In this study, we show that H_2_O_2_ induces intracellular ROS in human airway epithelial cells (BEAS-2B and NHBE cells) and enhances IL-1β-induced IL-6 and CXCL8 expression via increased p65 and Brd4 promoter association at κB sites in the native gene promoters. This is in conjunction with increases in histone H3 acetylation at these κB sites. The BET inhibitors JQ1 and PFI-1 both suppressed IL-1β- and IL-1β/H_2_O_2_-induced IL-6 and CXCL8 protein and mRNA expression in a concentration-dependent manner. Knockdown studies revealed that IL-6 and CXCL8 release is significantly reduced in Brd4, but not Brd2, depleted cells. Furthermore, H_2_O_2_-enhanced IL-1β-induced recruitment of p65 and Brd4 to the *IL-6* and *IL-8* promoters is abolished in JQ1-, but not in JQ1(-)-, pre-treated cells.

COPD primarily affects lungs; however, it now recognised a disease with organ-specific characteristics and systemic manifestations such as chronic inflammation [53]–[55]. We have analysed the expression of two of the most important pro-inflammatory cytokines, IL-6 and CXCL8, that are elevated in plasma, BAL fluids and sputum of COPD patients and whose expression correlates with disease severity [56]–[58]. These cytokines are regulated by the NF-κB signalling pathway which also orchestrates many aspects of the cellular immune response, apoptosis, differentiation and inflammation [59], [60]. High concentrations of H_2_O_2_ are found in breathe condensates of COPD patients and this has been linked to disease pathophysiology [61], [62]. For example, H_2_O_2_ can induce the release of potent chemotactic factors such as CXCL8 from epithelial cells [63] by resulting in increased hyper-acetylation at the *IL-8* promoter via alteration in HDAC/HAT balance [64].

NF-κB is a redox-sensitive transcription factor that converts oxidative stress signals into changes in gene expression associated with diverse cellular activities including inflammation [65]. NF-κB activation requires translocation of the NF-κB p65/p50 dimer into the nucleus where it binds to promoter regions of inflammatory genes forming a transcriptional activator complex [22], [66]. Optimal induction of NF-κB activity also requires post-translation modification of p65 e.g. phosphorylation, acetylation and ubiquitination, which modulates DNA binding and transcriptional activity. For example, acetylation of p65/RelA at Lysine-310 (K310) is essential for optimal NF-κB transcriptional activity [28], [30], [67]. Acetylated p65 provides a docking site for Brd4 and may be an important target in the ability of BET inhibitors to suppress inflammation [30]. In the present study, we demonstrated that p65 is acetylated within the nucleus following stimulation of cells with IL-1β and H_2_O_2_ although this did not affect the degree of p65:Brd4 association in the nucleus under any of the conditions tested. This data is in contrast to that observed in A549 lung adenocarcinoma-like cells where TNFα stimulation was essential for p65:Brd4 complex formation [30]. Our data suggests that in bronchial airway epithelial cells that Brd4 and p65 may be part of a pre-formed transcriptional complex.

An interesting observation made in this study is that the recruitment of Brd4 is associated with enhanced pan-acetylation of histone H3 but not of H4 at the *IL-6* and *IL-8* promoters confirming previous data [68]–[70]. Although, H_2_O_2_ alone was unable to induce H3 acetylation directly in BEAS-2B cells, it slightly enhanced the histone acetylation seen with IL-1β alone. This may reflect either a direct effect on localised HAT activity or a reduction in HDAC activity as previously reported in BEAS-2B cells [37]. This will result in an alteration in the local chromatin environment/structure which will modulate transcription factor association with promoter regions [71]. Future studies utilising DNase1 hypersensitivity assays, for example, may reveal changes in local chromatin structure following H_2_O_2_ treatment of cells which will enable greater recruitment of pTEFb by Brd4 and subsequent elongation of inflammatory gene expression by RNA polymerase II [72].

The chemically distinct bromodomain inhibitors (JQ1 and PFI-1) decreased recruitment of both Brd4 and p65 to the *IL-6* and *IL-8* promoters and reduced IL-6 and CXCL8 protein release from BEAS-2Bs and NHBE cells. This confirms data from Nicodeme and colleagues who reported that I-BET disrupts the interaction of bromodomain proteins and acetylated histones at the *IL-6* promoter in LPS-stimulated macrophages [25]. Inhibition of Brd4 either by JQ1 or knockdown reduced both IL-1β- and IL-1β/H_2_O_2_-induced IL-6 and CXCL8 expression by ∼80% whereas JQ1 completely blocked p65 DNA binding and subsequent Brd4 recruitment at the κB sites studied. AS602868, an IKK2 inhibitor, almost completely abrogated IL-1β- and IL-1β/H_2_O_2_-induced IL-6 and CXCL8 protein and mRNA expression confirming a key role for NF-κB in the transcriptional control of these genes. This suggests that other κB sites within the *IL-6* and *IL-8* promoters or enhancers may play a role in gene expression or that other inflammatory signalling pathways may modify the effect of JQ1 at the transcriptional and/or translational level. Future studies using ChIP-seq may address this. The failure to completely suppress CXCL8 gene expression may be important clinically as total suppression of the innate immune response may lead to opportunistic infections. The ability of JQ1 to dampen rather than ablate the immune/inflammatory response may therefore be of benefit in the treatment of patients with a chronic inflammatory disease. It will be of interest to determine whether JQ1 protects other innate immune genes in epithelial cells following stimulation with IL-1β or IL-1β/H_2_O_2_.

This is the first report to our knowledge that demonstrates a repressive effect of JQ1 and PFI-1 on IL-1β-induced CXCL8 expression. Previous studies [25], [73] have showed that CXCL1 mRNA, the murine equivalent of CXCL8, was unaffected by I-BET in mouse macrophages at baseline or after LPS-stimulation. However, Huang et al. [30] showed that the Brd4 depletion in LPS-stimulated THP-1 impaired CXCL8 expression. In our study Brd2 depletion in BEAS-2B cells had no effect on inflammatory cytokine expression. In contrast, Belkina *et al*. have shown that, in addition to Brd4, LPS-induced inflammation in murine bone marrow-derived macrophages (BMDMs) is mediated via Brd2 which binds to the *IL-6* promoter [73]. This difference may be species or cell type specific. ChIP-seq analysis indicates that Brd4 co-localizes with RNA pol II at both enhancers and promoters of all active genes in primary human CD4+ T cells. This interaction is disrupted upon JQ1 treatment leading to reduced lineage-specific gene expression and acetylated histone-bromodomain association [27]. The study implies that Brd4 could be used as a tool to identify active promoters and enhancers in a genome-wide manner in human epithelial cells. JQ1 has also been studied in NUT (Nuclear protein in testis) midline carcinoma (NMC) mouse model and various cancer cell lines, showing inhibition of cell proliferation and differentiation [31], [33]. Furthermore, *Brd4* is believed to be an inherited susceptibility gene for breast cancer progression and metastasis. It has been shown that deletion of Brd4 proline-rich domain at C-terminal results in a loss of contact inhibition and changed in cell morphology from epithelial to a mesenchymal-like phenotype [74].

There are some limitations to this study. Firstly, our data shows a difference in IL-6 and CXCL8 protein release in response to IL-1β and to IL-1β+H_2_O_2_ in the presence and absence of JQ1 suggesting that whatever effect IL-1β has on Brd4 function this is modified at least to some extent by the presence of H_2_O_2_ particularly in primary cells. However, we are unable to rule out the possibility that H_2_O_2_ stimulation modifies other aspects of p65 activity. Analysis of the effects of Brd4 knockdown on IL-1β- and H_2_O_2_-stimulated cells on mediator protein, mRNA and promoter ChIP analysis would help resolve this issue. Secondly, we show a differential effect of BET mimics on IL-6 and CXCL8 protein release compared with mRNA expression data. This indicates a degree of disconnect in both BEAS-2B and primary epithelial cells. Additional experiments, using combinations of BET protein knockdowns and pharmacological inhibition, should be performed to examine non-histone-based mechanisms of translational control and secretion.

In summary, we have shown that IL-1β and IL-1β/H_2_O_2_ can enhance recruitment of p65 and Brd4 to the native *IL-6* and *IL-8* promoters in epithelial cells. Inhibition of Brd4 by the structurally distinct bromodomain inhibitors JQ1 and PFI-1 and by genetic knockdown led to a reduction in *IL-6*/*-8* mRNA and protein expression. This was associated with an attenuation of p65 and Brd4 binding to the native *IL-6* and *IL-8* promoters. The specificity and long-term side effects of bromodomain inhibitors needs to be evaluated if these agents are to be used in chronic inflammatory diseases such as COPD and potentially in the progression to lung cancer [75]. Despite these concerns, our findings suggest that inhibition of Brd4 may have therapeutic potential in inflammatory diseases where oxidative stress and NF-κB activation is present.
